# Supplementary material for: A Tripartite Efflux System Affects Flagellum Stability in Helicobacter pylori
Source: Int J Mol Sci. 2022 Oct 1;23(19):11609. doi: 10.3390/ijms231911609 (PMC9570263; doi:10.3390/ijms231911609)
Supplement: Supplementary file 1 [file ijms-23-11609-s001.zip › ijms-1928327-supplementary.pdf]

Table S1. *Helicobacter* genomes examined for homologs of *H. pylori* G27 genes.

Helicobacter species with flagellar sheaths		
**species and strain**	**sequence status**	**reference**
Helicobacter acinonychis Sheeba	finished	[1]
Helicobacter ailurogastricus ASB7	incomplete	[2]
Helicobacter anseris MIT 04-9362	incomplete	
Helicobacter aurati isolate 137778_3	incomplete	[3]
Helicobacter baculiformis isolate 427351_3	incomplete	[4]
Helicobacter bilis AAQJH	finished	[5]
Helicobacter bizzozeronii CIII-1	finished	[6]
Helicobacter brantae MIT 04-9366	incomplete	[7]
Helicobacter canis NCTC 12740	finished	[8]
Helicobacter cetorum MIT 99-5656	finished	[9]
Helicobacter cholecystus ERZ467480	incomplete	[10]
Helicobacter cinaedi ATCC BAA-847	finished	[11]
Helicobacter cynogastricus 329937_4	incomplete	[12]
Helicobacter equorum 361872_4	incomplete	[13]
Helicobacter felis ATCC 49179	finished	[14]
Helicobacter fennelliae MRY-12-0050	finished	[11]
Helicobacter heilmannii ASB1.4	finished	[15]
Helicobacter hepaticus ATCC 51449	finished	[16]
Helicobacter himalayensis YS1	finished	[17]
Helicobacter jaachi MIT 09-6949	finished	[18]
Helicobacter japonicus MIT 01-6451	incomplete	[19]
Helicobacter labetoulli 48519	incomplete	[7]
Helicobacter macacae MIT 99-5501	finished	[20]
Helicobacter magdeburgensis MIT 96-1001	finished	[21]
Helicobacter marmotae 152490_3	incomplete	[22]
Helicobacter muridarum ST1	incomplete	[23]
Helicobacter mustelae 12198	finished	[24]
Helicobacter pametensis ATCC 51478	incomplete	[25]
Helicobacter rappini 95150_3	incomplete	[26]
Helicobacter saguini MIT 97-6194	finished	[27]
Helicobacter salomonis 56878_5	incomplete	[28]
Helicobacter suis HS1	incomplete	[29]
Helicobacter trogontum ATCC 700114	finished	[30]
Helicobacter typhlonius MIT 97-6810	finished	[31]

Helicobacter species with unsheathed flagella		
**species and strain**	**sequence status**	**[7]**
Helicobacter apodemus SCJK1	finished	[32]
Helicobacter burdigaliensis CNRCH 2005/566H	incomplete	[7]
Helicobacter canadensis MIT 98-5491	finished	[33]
Helicobacter ganmani MIT 99-5101	incomplete	[7]
Helicobacter mesocricetorum 87012_3	incomplete	[34]

Helicobacter pullorum MIT 98-5489	incomplete	[35]
Helicobacter rodentium ATCC 700285	incomplete	[36]
Helicobacter valdiviensis WBE14	incomplete	[37]
Helicobacter winghamensis ATCC BAA-430	finished	[38]

Table S4. Primers used in this study.

**primer ID #**	**primer name**	**sequence**
**P117**	HP1489 US_F	CATCAAAAACGCGGTGGA
**P118**	HP1489 US_R	GCTAGCATAATCGAATTCCTCGAGAGGAACTCC ATCAACAGCGCT
**P119**	HP1489 DS_F	CTCGAGGAATTCGATTATGCTAGCGCTTATAAA TACATTGTTTCATTAGCG
**P120**	HP1489 DS_R	CTTAGGGCTAAGCTCACCACC
**P129**	BamHI 1491 pr F	GGATCCTAATGGGCTTGCTTGAAC
**P130**	1491 promoter R	GAGGGTTGTTTTTTTCATACCGTCATAAAAACCTTATTC
**P131**	1489 gene F	GAATAAGGTTTTTATGACGGTATGAAAAAAACAACCCTC
**P132**	1489 gene R	CTCGAGTTAATAAACAAATTCATAAAATAA
**P139**	HP1488 US F	GAAGACATGATCCCTAGTTGGTTT-3′
**P140**	HP1488 US R	GCTAGCGATTCGATCCTCGAGCACTTCAGCCTT AGGGCG
**P141**	HP1488 DS F	CTCGAGGATCGAATCGCTAGCGAGTTTAGGGTG GGTAAGGAATTT
**P142**	HP1488 DS R	CCACTTGGTATTTGATTTGAAGTG
**P177**	1487 US F	AAGCGCGATGAAGCCTAT
**P178**	1487 US R	GCTAGCGATTCGATCCTCGAGCTTGTCTTGTAA AACCCATGC
**P179**	1486 DS F	CTCGAGGATCGAATCGCTAGCTTGAATCAAATG CATGCG
**P180**	1486 DS R	AAAAACGCTTGCAAATTTTC

References

1. Eppinger, M.; Baar, C.; Linz, B.; Raddatz, G.; Lanz, C.; Keller, H.; Morelli, G.; Gressmann, H.; Achtman, M.; Schuster, S.C. Who ate whom? Adaptive *Helicobacter* genomic changes that accompanied a host jump from early humans to large felines. *PLoS Genet* **2006**, *2*, e120, doi:10.1371/journal.pgen.0020120.eor.
2. Joosten, M.; Linden, S.; Rossi, M.; Tay, A.C.; Skoog, E.; Padra, M.; Peters, F.; Perkins, T.; Vandamme, P.; Van Nieuwerburgh, F.; et al. Divergence between the highly virulent zoonotic pathogen *Helicobacter heilmannii* and its closest relative, the low-virulence "*Helicobacter ailurogastricus*" sp. nov. *Infect Immun* **2016**, *84*, 293-306, doi:10.1128/IAI.01300-15.
3. Patterson, M.M.; Schrenzel, M.D.; Feng, Y.; Xu, S.; Dewhirst, F.E.; Paster, B.J.; Thibodeau, S.A.; Versalovic, J.; Fox, J.G. *Helicobacter aurati* sp. nov., a urease-positive *Helicobacter* species cultured from gastrointestinal tissues of Syrian hamsters. *J Clin Microbiol* **2000**, *38*, 3722-3728.
4. Baele, M.; Decostere, A.; Vandamme, P.; Van den Bulck, K.; Gruntar, I.; Mehle, J.; Mast, J.; Ducatelle, R.; Haesebrouck, F. *Helicobacter baculiformis* sp. nov., isolated from feline stomach mucosa. *Int J Syst Evol Microbiol* **2008**, *58*, 357-364, doi:10.1099/ijs.0.65152-0.

5. Fox, J.G.; Yan, L.L.; Dewhirst, F.E.; Paster, B.J.; Shames, B.; Murphy, J.C.; Hayward, A.; Belcher, J.C.; Mendes, E.N. *Helicobacter bilis* sp. nov., a novel *Helicobacter* species isolated from bile, livers, and intestines of aged, inbred mice. *J Clin Microbiol* **1995**, *33*, 445-454.
6. Schott, T.; Rossi, M.; Hanninen, M.L. Genome sequence of *Helicobacter bizzozeronii* strain CIII-1, an isolate from human gastric mucosa. *J Bacteriol* **2011**, *193*, 4565-4566, doi:10.1128/JB.05439-11.
7. Berthenet, E.; Benejat, L.; Menard, A.; Varon, C.; Lacomme, S.; Gontier, E.; Raymond, J.; Boussaba, O.; Toulza, O.; Ducournau, A.; et al. Whole-genome sequencing and bioinformatics as pertinent tools to support Helicobacteracae taxonomy, based on three strains suspected to belong to novel *Helicobacter* species. *Front Microbiol* **2019**, *10*, 2820, doi:10.3389/fmicb.2019.02820.
8. Stanley, J.; Linton, D.; Burnens, A.P.; Dewhirst, F.E.; Owen, R.J.; Porter, A.; On, S.L.; Costas, M. *Helicobacter canis* sp. nov., a new species from dogs: an integrated study of phenotype and genotype. *J Gen Microbiol* **1993**, *139*, 2495-2504, doi:10.1099/00221287-139-10-2495.
9. Harper, C.G.; Feng, Y.; Xu, S.; Taylor, N.S.; Kinsel, M.; Dewhirst, F.E.; Paster, B.J.; Greenwell, M.; Levine, G.; Rogers, A.; et al. *Helicobacter cetorum* sp. nov., a urease-positive *Helicobacter* species isolated from dolphins and whales. *J Clin Microbiol* **2002**, *40*, 4536-4543, doi:10.1128/jcm.40.12.4536-4543.2002.
10. Franklin, C.L.; Beckwith, C.S.; Livingston, R.S.; Riley, L.K.; Gibson, S.V.; Besch-Williford, C.L.; Hook, R.R., Jr. Isolation of a novel *Helicobacter* species, *Helicobacter cholecystus* sp. nov., from the gallbladders of Syrian hamsters with cholangiofibrosis and centrilobular pancreatitis. *J Clin Microbiol* **1996**, *34*, 2952-2958.
11. Totten, P.A.; Fennell, C.L.; Tenover, F.C.; Wezenberg, J.M.; Perine, P.L.; Stamm, W.E.; Holmes, K.K. *Campylobacter cinaedi* (sp. nov.) and *Campylobacter fennelliae* (sp. nov.): two new *Campylobacter* species associated with enteric disease in homosexual men. *J Infect Dis* **1985**, *151*, 131-139, doi:10.1093/infdis/151.1.131.
12. Van den Bulck, K.; Decostere, A.; Baele, M.; Vandamme, P.; Mast, J.; Ducatelle, R.; Haesebrouck, F. *Helicobacter cynogastricus* sp. nov., isolated from the canine gastric mucosa. *Int J Syst Evol Microbiol* **2006**, *56*, 1559-1564, doi:10.1099/ijs.0.63860-0.
13. Moyaert, H.; Decostere, A.; Vandamme, P.; Debruyne, L.; Mast, J.; Baele, M.; Ceelen, L.; Ducatelle, R.; Haesebrouck, F. *Helicobacter equorum* sp. nov., a urease-negative *Helicobacter* species isolated from horse faeces. *Int J Syst Evol Microbiol* **2007**, *57*, 213-218, doi:10.1099/ijs.0.64279-0.
14. Arnold, I.C.; Zigova, Z.; Holden, M.; Lawley, T.D.; Rad, R.; Dougan, G.; Falkow, S.; Bentley, S.D.; Muller, A. Comparative whole genome sequence analysis of the carcinogenic bacterial model pathogen *Helicobacter felis*. *Genome Biol Evol* **2011**, *3*, 302-308, doi:10.1093/gbe/evr022.
15. Smet, A.; Flahou, B.; D'Herde, K.; Vandamme, P.; Cleenwerck, I.; Ducatelle, R.; Pasmans, F.; Haesebrouck, F. *Helicobacter heilmannii* sp. nov., isolated from feline gastric mucosa. *Int J Syst Evol Microbiol* **2012**, *62*, 299-306, doi:10.1099/ijs.0.029207-0.
16. Suerbaum, S.; Josenhans, C.; Sterzenbach, T.; Drescher, B.; Brandt, P.; Bell, M.; Droge, M.; Fartmann, B.; Fischer, H.P.; Ge, Z.; et al. The complete genome sequence of the carcinogenic bacterium *Helicobacter hepaticus*. *Proc Natl Acad Sci U S A* **2003**, *100*, 7901-7906, doi:10.1073/pnas.1332093100.
17. Hu, S.; Jin, D.; Lu, S.; Liu, S.; Zhang, J.; Wang, Y.; Bai, X.; Xiong, Y.; Huang, Y.; Xu, H.; et al. *Helicobacter himalayensis* sp. nov. isolated from gastric mucosa of *Marmota himalayana*. *Int J Syst Evol Microbiol* **2015**, *65*, 1719-1725, doi:10.1099/ijs.0.000163.
18. Shen, Z.; Feng, Y.; Sheh, A.; Everitt, J.; Bertram, F.; Paster, B.J.; Fox, J.G. Isolation and characterization of a novel *Helicobacter* species, *Helicobacter jaachi* sp. nov., from common marmosets (*Callithrix jaachus*). *J Med Microbiol* **2015**, *64*, 1063-1073, doi:10.1099/jmm.0.000113.
19. Shen, Z.; Feng, Y.; Muthupalani, S.; Sheh, A.; Cheaney, L.E.; Kaufman, C.A.; Gong, G.; Paster, B.J.; Fox, J.G. Novel *Helicobacter* species *H. japonicum* isolated from laboratory mice from Japan

induces typhlocolitis and lower bowel carcinoma in C57BL/129 IL10-/- mice. *Carcinogenesis* **2016**, *37*, 1190-1198, doi:10.1093/carcin/bgw101.

20. Fox, J.G.; Boutin, S.R.; Handt, L.K.; Taylor, N.S.; Xu, S.; Rickman, B.; Marini, R.P.; Dewhirst, F.E.; Paster, B.J.; Motzel, S.; et al. Isolation and characterization of a novel helicobacter species, "*Helicobacter macacae*," from rhesus monkeys with and without chronic idiopathic colitis. *J Clin Microbiol* **2007**, *45*, 4061-4063, doi:10.1128/JCM.01100-07.
21. Traverso, F.R.; Bohr, U.R.; Oyarzabal, O.A.; Rohde, M.; Clarici, A.; Wex, T.; Kuester, D.; Malfertheiner, P.; Fox, J.G.; Backert, S. Morphologic, genetic, and biochemical characterization of *Helicobacter magdeburgensis*, a novel species isolated from the intestine of laboratory mice. *Helicobacter* **2010**, *15*, 403-415, doi:10.1111/j.1523-5378.2010.00770.x.
22. Fox, J.G.; Shen, Z.; Xu, S.; Feng, Y.; Dangler, C.A.; Dewhirst, F.E.; Paster, B.J.; Cullen, J.M. *Helicobacter marmotae* sp. nov. isolated from livers of woodchucks and intestines of cats. *J Clin Microbiol* **2002**, *40*, 2513-2519, doi:10.1128/jcm.40.7.2513-2519.2002.
23. Lee, A.; Phillips, M.W.; O'Rourke, J.L.; Paster, B.J.; Dewhirst, F.E.; Fraser, G.J.; Fox, J.G.; Sly, L.I.; Romaniuk, P.J.; Trust, T.J.; et al. *Helicobacter muridarum* sp. nov., a microaerophilic helical bacterium with a novel ultrastructure isolated from the intestinal mucosa of rodents. *Int J Syst Bacteriol* **1992**, *42*, 27-36, doi:10.1099/00207713-42-1-27.
24. Fox, J.G.; Cabot, E.B.; Taylor, N.S.; Laraway, R. Gastric colonization by *Campylobacter pylori* subsp. mustelae in ferrets. *Infect Immun* **1988**, *56*, 2994-2996.
25. Dewhirst, F.E.; Seymour, C.; Fraser, G.J.; Paster, B.J.; Fox, J.G. Phylogeny of *Helicobacter* isolates from bird and swine feces and description of *Helicobacter pametensis* sp. nov. *Int J Syst Bacteriol* **1994**, *44*, 553-560, doi:10.1099/00207713-44-3-553.
26. Dewhirst, F.E.; Fox, J.G.; Mendes, E.N.; Paster, B.J.; Gates, C.E.; Kirkbride, C.A.; Eaton, K.A. '*Flexispira rappini*' strains represent at least 10 *Helicobacter* taxa. *Int J Syst Evol Microbiol* **2000**, *50 Pt 5*, 1781-1787, doi:10.1099/00207713-50-5-1781.
27. Shen, Z.; Mannion, A.; Whary, M.T.; Muthupalani, S.; Sheh, A.; Feng, Y.; Gong, G.; Vandamme, P.; Holcombe, H.R.; Paster, B.J.; et al. *Helicobacter saguini*, a novel *Helicobacter* isolated from cotton-top tamarins with ulcerative colitis, has proinflammatory properties and induces typhlocolitis and dysplasia in gnotobiotic IL-10-/- mice. *Infect Immun* **2016**, *84*, 2307-2316, doi:10.1128/IAI.00235-16.
28. Jalava, K.; Kaartinen, M.; Utriainen, M.; Happonen, I.; Hanninen, M.L. *Helicobacter salomonis* sp. nov., a canine gastric *Helicobacter* sp. related to *Helicobacter felis* and *Helicobacter bizzozeronii*. *Int J Syst Bacteriol* **1997**, *47*, 975-982, doi:10.1099/00207713-47-4-975.
29. Baele, M.; Decostere, A.; Vandamme, P.; Ceelen, L.; Hellemans, A.; Mast, J.; Chiers, K.; Ducatelle, R.; Haesebrouck, F. Isolation and characterization of *Helicobacter suis* sp. nov. from pig stomachs. *Int J Syst Evol Microbiol* **2008**, *58*, 1350-1358, doi:10.1099/ijs.0.65133-0.
30. Mendes, E.N.; Queiroz, D.M.; Dewhirst, F.E.; Paster, B.J.; Moura, S.B.; Fox, J.G. *Helicobacter trogontum* sp. nov., isolated from the rat intestine. *Int J Syst Bacteriol* **1996**, *46*, 916-921, doi:10.1099/00207713-46-4-916.
31. Frank, J.; Dingemanse, C.; Schmitz, A.M.; Vossen, R.H.; van Ommen, G.J.; den Dunnen, J.T.; Robanus-Maandag, E.C.; Anvar, S.Y. The complete genome sequence of the murine pathobiont *Helicobacter typhlonius*. *Front Microbiol* **2015**, *6*, 1549, doi:10.3389/fmicb.2015.01549.
32. Jeon, W.J.; Dong, H.J.; Shin, J.H.; Kim, I.Y.; Ho, H.; Oh, S.H.; Yoon, Y.M.; Choi, Y.K.; Suh, J.G.; Nam, K.H.; et al. *Helicobacter apodemus* sp. nov., a new *Helicobacter* species identified from the gastrointestinal tract of striped field mice in Korea. *J Vet Sci* **2015**, *16*, 475-481, doi:10.4142/jvs.2015.16.4.475.

33. Loman, N.J.; Snyder, L.A.; Linton, J.D.; Langdon, R.; Lawson, A.J.; Weinstock, G.M.; Wren, B.W.; Pallen, M.J. Genome sequence of the emerging pathogen *Helicobacter canadensis*. *J Bacteriol* **2009**, *191*, 5566-5567, doi:10.1128/JB.00729-09.
34. Simmons, J.H.; Riley, L.K.; Besch-Williford, C.L.; Franklin, C.L. *Helicobacter mesocricetorum* sp. nov., a novel *Helicobacter* isolated from the feces of Syrian hamsters. *J Clin Microbiol* **2000**, *38*, 1811-1817.
35. Stanley, J.; Linton, D.; Burnens, A.P.; Dewhirst, F.E.; On, S.L.; Porter, A.; Owen, R.J.; Costas, M. *Helicobacter pullorum* sp. nov.-genotype and phenotype of a new species isolated from poultry and from human patients with gastroenteritis. *Microbiology* **1994**, *140 (Pt 12)*, 3441-3449, doi:10.1099/13500872-140-12-3441.
36. Shen, Z.; Fox, J.G.; Dewhirst, F.E.; Paster, B.J.; Foltz, C.J.; Yan, L.; Shames, B.; Perry, L. *Helicobacter rodentium* sp. nov., a urease-negative *Helicobacter* species isolated from laboratory mice. *International journal of systematic bacteriology* **1997**, *47*, 627-634, doi:10.1099/00207713-47-3-627.
37. Collado, L.; Jara, R.; Gonzalez, S. Description of *Helicobacter valdiviensis* sp. nov., an Epsilonproteobacteria isolated from wild bird faecal samples. *Int J Syst Evol Microbiol* **2014**, *64*, 1913-1919, doi:10.1099/ijs.0.057141-0.
38. Melito, P.L.; Munro, C.; Chipman, P.R.; Woodward, D.L.; Booth, T.F.; Rodgers, F.G. *Helicobacter winghamensis* sp. nov., a novel *Helicobacter* sp. isolated from patients with gastroenteritis. *J Clin Microbiol* **2001**, *39*, 2412-2417, doi:10.1128/JCM.39.7.2412-2417.2001.
